# Barriers to overcoming immunotherapy resistance in glioblastoma

**DOI:** 10.3389/fmed.2023.1175507

**Published:** 2023-05-18

**Authors:** Julia S. Gillette, Elaina J. Wang, Richard S. Dowd, Steven A. Toms

**Affiliations:** Departments of Neurosurgery, The Warren Alpert Medical School of Brown University, Providence, RI, United States

**Keywords:** gliobalstoma, immunotherapy, checkpoint inhibitors, vaccine, chimeric antigen receptor (CAR) T cells, virotherapy, resistance

## Abstract

Glioblastoma multiforme (GBM) is the most common malignant primary brain tumor, known for its poor prognosis and high recurrence rate. Current standard of care includes surgical resection followed by combined radiotherapy and chemotherapy. Although immunotherapies have yielded promising results in hematological malignancies, their successful application in GBM remains limited due to a host of immunosuppressive factors unique to GBM. As a result of these roadblocks, research efforts have focused on utilizing combinatorial immunotherapies that target networks of immune processes in GBM with promising results in both preclinical and clinical trials, although limitations in overcoming the immunosuppressive factors within GBM remain. In this review, we aim to discuss the intrinsic and adaptive immune resistance unique to GBM and to summarize the current evidence and outcomes of engineered and non-engineered treatments targeted at overcoming GBM resistance to immunotherapy. Additionally, we aim to highlight the most promising strategies of targeted GBM immunotherapy combinatorial treatments and the insights that may directly improve the current patient prognosis and clinical care.

## 1. Introduction

Glioblastoma (GBM) is the most common malignant primary central nervous system (CNS) tumor in the United States, representing 14.3% of all tumors, 49.1% of malignant tumors, and 58.4% of gliomas (1–3). While the 5-year survival rate for all malignant brain tumors combined is 36%, the unique intrinsic and adaptive immune resistance that characterizes GBM translates to an even lower 5-year survival rate of 5.7% in a DCVax control population and 5% in TTF EF-14 trial, with a median survival of 14.7–16.5 months in DCVax and TTF trial control (4, 5). The standard of care for newly diagnosed GBM includes maximal surgical resection followed by adjuvant combinatorial chemotherapy and radiation and subsequent temodar combined with temodar-tumor treating fields (2, 6, 7). Almost all patients (~90%) experience tumor recurrence and there is no established standard of care for recurrent glioblastoma (rGBM) other than supportive and palliative care (1). Although repeating radiotherapy and chemotherapy or the use of anti-angiogenic drugs such as bevacizumab remain options for certain patients, the 2-year survival for rGBM remains at 26% (8–10).

There is significant focus on creating novel multimodal therapies to target the unique biological characteristics and immunosuppressive factors unique to GBM (11). Recently, clinical trials have shown moderate improvement in median overall survival of 20.9 months from 16.0 months within Tumor-Treating Fields plus temozolomide chemotherapy treatment (TTFields-temozolomide) compared to temozolomide alone, respectively (4). Targeted immune therapies, most notably: signaling pathway inhibitors, checkpoint inhibitors, and Chimeric antigen receptor (CAR)-T cell therapy, have significantly improved the treatment of various hematologic malignancies, with over 50 approved therapies within the last decade (12). These immune therapies harness the patient’s own immune response to target specific tumor cells. Recent studies have focused on applying the success of immune therapies within GBM populations; a recent single patient case report demonstrated significant GBM tumor regression following IL13Rα2-targeted chimeric antigen receptor (CAR)–engineered T cells therapy administration, with subsequent host-immune response increases sustaining 7.5 months post-treatment (13).

Aside from CAR-T cells, a variety of other immune strategies have been employed in GBM. A multitude of immune checkpoint inhibitor trials have been attempted but have thus far failed to move the needle on survival (14, 15). Single and multi-epitope peptide vaccines have in general not impacted survival, but the surviving long peptide vaccine (SurVaxM) has had an impact in recurrent GBM in early phase trials and is moving toward phase 3 trials in malignant glioma (16). Most recently, the strategy of personalized dendritic cell vaccine has completed a Phase 3 trial with modest improvements in survival vs. an external control group (17). Although these reports illustrates potential, the widespread success of immunotherapies within hematologic and solid malignancies has failed to translate to larger GBM trials, and to date no immunotherapies have been approved for glioblastoma (18, 19). The lack of success in clinical trials to improve the SOC for glioblastoma underlines the importance of further understanding the intrinsic and adaptive factors of GBM that encapsulate the aggressive nature of this tumor.

There are several reasons that the therapies applied in hematological malignancies have not translated to the same level of success in GBM. GBM’s many immunosuppressive properties can be divided into both intrinsic and adaptive factors. Intrinsic factors of GBM include multiple areas of immunosuppression through intratumoral heterogeneity (ITH), qualitative and quantitative T-cell immune dysfunction (20), tumor-mediated immune sequestering of T-cells within the bone marrow (21), and the immune distinct microenvironment of the central nervous system (22–24). Adaptive factors of GBM include plasticity of Glioblastoma stem-like cells (GSCs), selection of resistant intratumoral populations, effect of concurrent steroid treatments (Dexamethasone) on immunotherapy (25) and adaptive genomic and epigenomic changes (26, 27) in recurrent glioblastoma that ultimately increase lethality. In this paper we discuss in detail the most recent advancements to the understanding of these intrinsic and adaptive factors of immunotherapy resistance within GBM. Additionally, we will discuss the latest engineered medicines, such as CD47, CSF1R, CD73, COX2, CCL2, IL6, and GITR inhibitors, and non-engineered medicines that possess the potential to overcome these clinical obstacles and improve prognosis for patients.

## 2. Intrinsic factors of resistance in GBM

### 2.1. Immunosuppression

The GBM microenvironment is known for its immunosuppressive properties (Supplementary Table 1). The clonal progression of tumor cells selects for highly proliferative and therapy-resistant clones that allows for the tumor to grow and evade immune responses and immunotherapies (28). It has been demonstrated that up to 50% of the GBM cells are comprised of tumor-associated macrophages (TAMs), 15% being microglia (MG) and 85% being monocyte-derived macrophages (MDMs) (29–31). It is currently understood that mesenchymal GBM has the highest percentage of microglia (CD68+) and bone marrow derived macrophages (CD68+) (32). While M2-similar TAMs are associated with higher graded tumors, M1-similar TAMs contain anti-tumor properties (33), illustrating the importance of characterizing the TAM microenvironment within each tumor (34, 35). Recently it has been demonstrated that chitinase-3-like 1 (CHI3L1), a protein complex upregulated in GBM, increases immunosuppression within the tumor microenvironment by increasing infiltration of MDMs and MG while additionally supporting TAM immunosuppression and subsequent residence to therapies (34).

One of the most impactful immunosuppressive mechanisms in GBM is the increased abundance of myeloid-derived suppressor cells (MDSC), cells of the innate immune system (36) that act to suppress cytotoxic T-cells and inhibit the memory ability of CD4+ cells (37, 38). The small molecule drug Sunitinib, a receptor tyrosine kinase inhibitor, has proven to be an effective treatment that targets MDSC and subsequently increases CD3+ and CD4+ microenvironment T-cells (38). T-cell dysfunction in GBM can also be attributed to the sequestering of T-cells within the bone marrow due to T-cell surface loss of S1P1 within the tumor microenvironment (21). Reversal of this sequestering through immunotherapy has shown to be an effective adjunctive therapy (21). Additionally, increased expression of CD8+ cells and CD163+ cells have demonstrated to be correlated with worse survival and prognosis due to lymphocyte immunosuppression (32, 39). Additionally, the GBM microenvironment includes glioma stem-like cells (GSCs) which evade immune therapies through multiple mechanisms including but not limited to the down regulation of MHC class I molecules, increasing Treg cells within the microenvironment, increasing TAM-produced TGF-β, and resulting down regulation of MHC II (40).

### 2.2. Inter and intra-tumoral heterogeneity

Glioblastoma inter- and intra-tumoral heterogeneity (ITH) is one of the primary intrinsic mechanisms explaining therapy resistance in GBM, ultimately resulting in hypermutation that creates clinical barriers (41). As seen with The Cancer Genome Atlas (TCGA), GBM’s inter-tumoral heterogeneity can act as a baseline of classification (42). Glioblastoma is a grade IV glioma, the most aggressive and deadly glioma. The World Health Organization previously classified GBM as primary or secondary (43), with primary GBM both beginning at and being diagnosed as grade IV. However, the updated WHO 2021 classification does not identify a secondary GBM as a GBM, and instead strictly defines GBM by IDH wildtype (44). Further classification of GBM is based on tumor transcription profile, genetic changes, and DNA methylation status; these classifications aid in the determination of appropriate therapies. Transcription profiles are commonly characterized by four subtypes: Proneural, Neural, Classical and Mesenchymal (42). The genetic alterations and molecular profiles characteristically altered in GBM are IDH-wildtype, TERT promoter, gain of chromosomes7, loss of chromosome 10, and EGFR amplification (44, 45). It should be noted that CDKN2A/B homozygous deletions is a strong indicator for poor prognosis and has been distinguished from Glioblastoma to be categorized as IDH-mutant astrocytoma: astrocytoma, IDH-mutant, CNS WHO grade IV (45). Classification based on genetic alterations are also primarily characterized by mutations in the PTEN (Phosphatase and tensin homolog) gene (46, 47). Research studying GBM subtypes based on DNA methylation status varies regarding methylation clustering, with most recent prediction mechanisms focusing on CpG promoter regions (46, 48).

Intra-tumoral heterogeneity refers to cellular differences within the tumor microenvironment (42). One of the main mechanisms of heterogeneity is transcriptional diversity, specifically in regards to oncogenic signaling, proliferation, immune signaling, and angiogenesis (49). Single-cell RNA sequencing (scRNA-seq) is a powerful tool that allows for the identification of heterogeneity within GBM tumors by identifying cell subpopulations, gene expression levels, and insight into the tumor microenvironment (49, 50). The classification system proposed by Verkaak et al. provides a framework for understanding intra-tumoral heterogeneity in regards to transcriptional variation in gene signatures: the Mesenchymal subtype corresponds primarily to pathways involved in immune response such as mutations in the NF-1 gene, the Proneural subtype primarily corresponds with cell-signaling and cell cycle regulation and involve the TP53, IDH-1, 1p/19q-codeletions, and PDGFR-A genes, the Neural subtype corresponds to nervous system signaling, and the Classical subtype is enriched in pathways such as Fatty Acid metabolism processes, nervous system processes, B Lymphocyte signaling within the immune system, and amplification of the EGFR gene (46). It should be noted that the World Health Organization reclassified Proneural subtypes in 2021. Since all proneural subtypes contain and IDH1 mutation, they are no longer considered GBM (51). Identifying these alterations within the tumor microenvironment using scRNA-seq and subsequent classification is essential when creating the optimal targeted treatment approach for each unique GBM tumor.

Single-cell RNA sequencing (scRNA-seq) similarly provides insight into the cancer stem cell diversity within a tumor micro-environment (49, 50, 52). GBM cells have been shown to dedifferentiate into cells with stem cell-like properties, increasing tumor plasticity and diversity (53). In response to treatments, some patients may experience tumors with a high tumor mutation load, where GBM tumors become diversely hypermutated. Although some of the phenotype (proteomic) changes in GBM occur because of genetic mutations, a large number of the phenotype changes in glioblastoma are secondary to epigenetic—histone methylation, transcriptome, RNA methylation, and protein alteration (54). For example, one recent study found that different types of GSCs are created at the tumor’s invasive edge more than the necrotic core in response to radiation therapy (55). These cells lose the CD133 protein and express the CD109 protein to become classified as mesenchymal subtype, a marker of increased tumor growth and differentiation via the YAP/TAZ pathway (55). Additionally, it has been found that the most invasive cells in a tumor edge express markers of migratory mesenchymal subtypes through epigenome, transcriptome, and proteome changes, such as CHI3L1, PDPN, FAM2OC, SERPINE and CD44 (56). The interest in exploring heterogeneity of cell phenotypes between the invasive edge and tumor core does not stop at GSCs. The invasive edge is histologically notable as “pseudopalisading” necrosis and includes highly proliferative cells that secrete VEGF and IL-8 and over-express HIF-1 (57). Interestingly, cells at the invasive edge vs. the core have differing gene expression patterns, with the invasive cells notably over-expressing the genes TNFRSF12A and CTGF, a TNF-related gene and cell-process gene, which are markers for growth and therapy-resistance (58, 59). As we see from these findings, GBM tumors are non-homogeneous; accurately characterizing and understanding the heterogeneity within the GBM tumor microenvironment is essential for executing the most efficacious combinatorial treatments.

### 2.3. Immune dysfunction

Immune dysfunction in GBM, primarily T-cell dysfunction, debilitates the anti-tumor immune network and further restricts the efficacy of immunotherapies. Immune-checkpoint inhibitors (ICPi), such as those targeting CTLA-4 and the PD-1 pathways, have been highly successful in increasing overall survival in many cancers such as melanoma and non-small cell lung cancer (60). Due to the aggressive immunosuppressive microenvironment of GBM, ICPi have yielded mixed or disappointing outcomes thus far within GBM. A recent open-label, phase III CheckMate 498 study showed that Nivolumab (NIVO), a human immunoglobulin G4 monoclonal antibody that inhibits PD-1, plus RT failed to increase overall survival in a sample size of 280 patients compared to RT + TMZ (*n* = 280) (14). Additionally, dendritic cell based therapeutic vaccines such as DCVax are required to initiate a targeted Tcell cellular immune response prior to ICPi therapy administration in order to maximize results (17, 61). However, recent evidence suggests increased efficacy of ICPi as a neoadjuvant therapy by increasing the local and systemic immune response in some patients. Specifically, a 2019 randomized, multi-institution clinical trial by Cloughesy et al. found that patients receiving neoadjuvant PD-1 blockade (pembrolizumab) showed increased gene expression responsible for T cell and interferon-γ and decreased expression of cell-cycle genes (62). Although these early results are encouraging, a more recent study in 2021 found that benefits from neoadjuvant PD-1 blockade failed to counteract immunosuppressive tumor associated macrophages found in rGBM (63). These findings highlights the importance of (1) further understanding the mechanism behind immune dysfunction within GMB, and (2) continual creation of highly-specific combinatorial therapies (60).

A recent mechanistic theory describing T-cell dysfunction has shown that HMOX1^+^ myeloid cells increase the signaling of anti-inflammatory cytokine IL-10 which depresses the natural functions of T cells (64). The same study found that blocking of the IFNgamma-JAK/STAT signaling pathway using the inhibitor Ruxolitinib decreased IL-10 signaling which partially resolved immunosuppression in a patient with rGBM (64). This partial rescue of the immunosuppressive microenvironment of dysfunctional t-cells suggests a possible adjuvant therapeutic target that could be used within combinatorial therapies.

Additionally, impaired lytic function of GBM tumor-infiltrating natural killer (NK) cells has been shown to be a mechanism of immune dysfunction in GBM (65). The same study showed that impairment of these NK cells is due to glioblastoma stem cell-NK cell interaction and subsequent αv integrin-mediated TGF-β activation. Further, the authors suggest that targeting of the αv integrin/TGF-β axis may provide a beneficial combinatorial therapy (65).

## 3. Adaptive factors of resistance in GBM

### 3.1. Selection of resistant intratumoral populations and changes in recurrent glioblastoma

GBM has also been shown to acquire mechanisms of secondary resistance. One of these mechanisms is through a uniquely aggressive diffuse infiltrative growth pattern, which is a main pathway of drug resistance within GBM, particularly to temozolomide, as well as recurrence at area of lesion (66). GBM diffusely disseminates so that complete surgical resection is impossible and subsequent radiotherapy and chemotherapy is necessary (67). Around 90% of GBM patients experience recurrence, and this recurrence is usually seen around the area of surgical resection margin (68). One mechanism for tumor lesion repair within GBM is that glioma cells utilize extra-long membrane protrusions, or “tumor microtubes” (TMs), to accomplish invasion, proliferation, and repopulation after treatment (67). A recent study illustrates that TM-connected glioma cells both heal and repair at the site of lesion as well as provide resistance against DNA alkylating agents like temozolomide (67).

The plasticity of Glioblastoma stem-like cells (GSCs) is a leading adaptive factor of GBM that contributes significantly to therapy resistance (69). GBM cancer stem cells have the ability to adapt and become more aggressive and resistant to radiation post initial radiotherapy. It was found that GSCs within mice activate IGF1R-dependent pathways that repairs damage and induces radioprotective abilities (70). Similarly, radiation induces protective autophagy within CD133+ GSCs and glioma cells, which has been shown to increase radioresistant properties after treatment by inducing cell death in these neoplastic cells (70, 71). A recent study illustrated that both the inhibition and induction of autophagy may be used as therapeutic approaches to high-grade malignant GBM (72). There is increasing evidence that autophagy inhibition can sensitize CD133+ to radiotherapy and chemotherapy, while the induction of autophagy can be beneficial by increasing cell apoptosis during treatment (73, 74). The use and balancing of autophagy inducers and inhibitors presents as a promising combinatorial therapy to target gamma-radiation resistance in GBM.

One of the most impactful breakthroughs in chemotherapy-treated GBM research to date is when Concomitant and Adjuvant Temozolomide following radiation therapy was shown to increase median OS by 2 months (4). However, unfortunately the widespread use and relative success of TMZ in GBM standard of care is met with tumor recurrence and resistance. One of the mechanisms of resistance to TMZ in the upregulation of DNA-repair mechanisms after treatment and the resulting evolutionary selective increase of stem-like CD133+ cell population, resistant GSCs. TMZ resistance has also been found to primarily be accomplished through DNA repair, increased production of GBM cell stemness, HDAC activity, and increased transcription factor mechanism (75). Current standards of care for GBM include surgical resection, radiotherapy, and adjuvant chemotherapy. However, Radiotherapy in combination with chemotherapy such as TMZ (RT-TMZ) has been shown to cause lymphopenia, drastically decrease CD4+ and CD8+ T cells, and increase functional Tregs, ultimately ensuring an immunosuppressive environment (76). More careful consideration of the chemotherapy-induced immunosuppressive effects or altering the timing of the concomitant use of chemotherapeutic and immune agents may be required as newer therapies emerge.

GBM treated with immune therapies has shown to evolutionally respond through pathways that select for immune-therapy resistant tumor cells. Epidermal growth factor receptor variant III (EGFRvIII) has become a target for immunotherapies by the utilization of Chimeric antigen receptor (CAR) T cells. While this immunotherapy has shown success as a hematological malignancy treatment (77), GMB tumors compensatory adapt by increasing immunosuppressive regulatory T cells and up-regulating expression of IDO1, PD-L1, and IL-10 after immunosuppressive molecules post CART-EGFRvIII infusion (78). Moreover, rGBMs post IL13Rα2-CAR T cells therapy resulted in IL13Rα2 antigen loss GBM variants that increased the tumor survival and proliferation (79). These single epitope therapies are likely to be overcome by the plasticity (80) and antigen drift (81) in GBM tumors, shifting the tumor to a treatment-resistant state.

### 3.2. Effect of steroids on immune response

Perioperative high-dose steroid therapy (dexamethasone) is considered an important aspect of GBM standard of care to reduce the increased cranial pressure and edema caused by brain tumors (82). Dexamethasone is a corticosteroid with relatively greater potency and ability to navigate past the blood brain barrier (83). Recent studies have investigated the impact that steroid treatments have on immunosuppression in GBM, both systemically and when used concurrently with immunotherapies.

Dexamethasone used concurrently with anti-PD-1 therapy was found to decrease OS in GBM patients in a dose-dependent manner through the decreasing of T-lymphocytes and lymphocyte functionality, and the reduction of myeloid and natural killer cells (25). Perisurgical steroid therapy is thought to contribute to CCR7 expression loss in T cells, which triggers the sequestering of T cells to the IL15-heavy bone marrow (84). Similarly, recent research determined that TTF-treated GBM patients and those treated with chemotherapy, along with increased dexamethasone doses (>4.1 mg per day) had significantly reduced OS compared with patients who received ⩽4.1 mg per day (85).

Concurrent dexamethasone therapy has been shown to significantly decrease the efficacy of the increasingly promising Intratumoral viral oncolytic immunotherapy, CAN-2409 (86). CAN-2409, a replication-deficient adenovirus, is injected intratumorally and induces both a local immune response and a systemic immune response. Although CAN-2409 has shown promising results in phase II clinical trials (87), its efficacy is greatly diminished when used in conjunction with continuous high-dose dexamethasone (86).

However, a recent study clarified that pre-surgical use of dexamethasone is beneficial in SOC practices but not when used adjunctly with immunotherapies (88). Further, the authors suggest that a decision to treat patients with immunotherapies can be guided by pre-steroid peripheral lymphocyte blood count levels at the time of diagnosis, with increased immunotherapy efficacy found in patients with a higher baseline lymphocyte count (88).

An emerging area of interest within GBM research is the use of corticosteroid-reducing agents. There have been studies suggesting that anti–vascular endothelial growth factor antibodies, specifically, bevacizumab and corticorelin acetate, may show potential as replacements for Dexamethasone (83). As a result, the Response Assessment in Neuro-Oncology (RANO) Working Group suggests the implementation of a corticosteroid response definition as a new endpoint within neuro-oncological clinical studies (83). These findings and proposals emphasize the increasing interest in balancing, or possibly replacing, Dexamethasone dosing for the treatment of tumor-induced edema with the use of immunotherapy in GBM patients.

## 4. Overcoming resistance with engineered medicines

Multiple immunotherapies have been created to target the intrinsic and adaptive resistance seen in GBM. Currently, these therapies include immune checkpoint inhibitor therapy, vaccination therapy, oncolytic virotherapy, adaptive t-cell therapies, and CAR-T therapies (89, 90). Here we describe emerging engineered medicines that aim to generate an anti-tumoral immune response against the uniquely aggressive GBM-mediated immunosuppression (Supplementary Table 2).

### 4.1. CD47 inhibitors

The microenvironment of GBM is characterized by high levels of myeloid cells, specifically macrophages and microglia, called tumor-associated macrophages (TAMs) (91). TAMs recruit growth factors and pro-survival cytokines within the tumor microenvironment that increases glioma proliferation (30). TAM levels are associated with grading of gliomas and prognosis (92). The majority of these TAMs (85%) are derived from the bone marrow and recruited to the location of the tumor while the remaining are resident microglia that innately provide anti-tumor properties (58). This presents two pathways for targeting TAMs: (1) targeting the infiltrating bone-marrow derived TAMs, and (2) restoration of the anti-tumor activity of locally resident microglia (90).

CD47 is highly expressed in GBM; GBM cells that express CD47 and bind to SIRPa expressed on myeloid cells restrict macrophages from phagocytosing GBM cells (10, 65, 90). Therefore, CD47-SIRPa axis blockades are a promising immunotherapy target. A recent study found that CD47 blockades used concurrently with temozolomide activated both the innate and adaptive immune response through the increase of phagocytosis and more efficient T-cell priming through APCs (93). Similarly, it was found that administration of anti-CD47 antibodies increased macrophage phagocytosis of both glioma cells and GSCs, with decreases in tumor growth and increased survival time in animal models (94). Another study found that the efficacy of anti-CD47 therapies are enhanced separately by both Irradiation and temozolomide administration by decreasing tumor growth and increasing survival times in mouse models (95). The authors illustrate that these results are similarly due to the increased macrophage/microglia-mediated phagocytosis of GBM (95).

### 4.2. CSF1R inhibitors

Colony stimulating factor (CSF-1) is needed for the survival of macrophages; inhibition of the CSF-1 receptor in GBM has showed promising results in the regression of existing tumors, blocking of tumor progression, and increased survival within both mouse models and human xenografts (96). A recent study found that the small molecule CSF-1R inhibitor PLX3397 inhibited growth of PDGFB-driven GBM models (97). However, the same study found that RAS-driven tumor growth was *accelerated* by the targeting of TAMs in early phases. The authors further found that these finding are due to different signaling of TAM survival by different subtypes of GBM: PDGFB-driven GBM induced activation of TAMs while mesenchymal RAS-driven GBM signal TAM survival through inflammation and angiogenic signaling, with RAS-driven GBM effectively targeted through the combination anti-TAM and angiogenesis (97). These findings reveal differences in TAMs activation and signaling between tumor sub-types and illustrate the importance of understanding the microenvironment of these subtypes to accurately design TAM-targeted therapies.

There has been evidence that tumor recurrence after CSF-1R blockade therapy is common (>50% within a mice model), characterized by a resensitization to CSF-1R inhibition in rGBM (33). In response to IL4, TAMs acquire the ability to upregulate expression of insulin-like growth factor 1 (IGF-1), which triggers the signaling of IGF-1R and PI3K, rendering the tumor resistant to CSF-1R inhibition (33, 98). However, the same study found that OS in CSF-1R resistant rGBM can be increased by IGF-1R or PI3K inhibition in conjunction with CSF-1R blockade therapy (33).

GBM often recurs after ionizing radiation treatment due to IR-induced myeloid cell signaling that increases TAM populations (99). CSF-1R inhibitor PLX3397 was found to not only sensitize GBM to radiation but also decreased the differentiation of cells into TAMs (99). A similar study confirmed RT-induced TAM population increases, with CSF-1R inhibitor BLZ-945 reversing this (100). Further, the authors found that while CSF-1R inhibitor BLZ-945 alone did not significantly increase OS, RT and the CSF-1R inhibition combined increased OS in mice models more than RT or BLZ-945 alone (100).

### 4.3. CD73 inhibitors

CD73 activity is understood to increase proliferation of GBM cells (101). CD73 macrophages are upregulated in GBM and this upregulation is often resistant to anti-PD-1 therapy (102). However, a recent study has shown that the combinatorial therapy comprising CD73 inhibitors and anti-PD-1 therapies improved OS in marine models (102). Additionally, CD73 is a regulator of epithelial-mesenchymal-like transition (EMT), which plays a role in GSC proliferation and infiltration (103). CD73 inhibition by phosphodiesterase inhibitor pentoxifylline was shown to decrease GSC survival *in vitro* (104). Further, CD73 inhibition or blockage of CD73 activity has been shown to accomplish GBM chemosensitization due to suppression of multiple drug associated protein 1 (Mrp1) (101).

### 4.4. COX2 inhibitors

Pro-inflammatory protein Cyclooxygenase-2 is upregulated and constitutionally expressed in GBM and is associated with increased aggressiveness, worse survival, and higher grade malignancies through immune evasion and consequent immunotherapy resistance within GBM (105–107). While not accurately described as an immunotherapy, COX2 inhibitors may be useful as a combinatorial therapy by increasing immunotherapy sensitization (108).

Tumor-promoting effects of COX-2 expression in GBM is mostly due to the prostaglandin E2 (PGE2) product which compounds tumor progression and both chemoresistance and radioresistance (105, 109). Further, the PGE2 product from COX-2 enhances GSC cloning and stemness through the MAPK signal cascade resulting in increasing inhibitor of differentiation 1 (Id1)-induced Wnt signaling pathways that generates this marked therapeutic resistance (110). The increased COX-2 expression in GBM has also been shown to correlate with vascular endothelial growth factor (VEGF), which in turn induces angiogenesis and increased blood supply to the tumor and resulting prognostic effects (111, 112).

COX-2 has gained significant interest as a therapeutic target for GBM. In a recent study using glioma cell lines, Three COX-2 inhibitors NS-398, Celecoxib and Meloxicam, have been shown to reduce GBM proliferation and increase radiosensitization when used before initial radiotherapy, independent of differing COX-2 expression levels (113). Additionally, NS-398 was found to decrease migration of tumor cells in a dose-dependent manner (106). Preclinical studies using animal GBM xenografts treated with NSAIDs and COXIBs have shown promising results and have illustrated an anti-seizure component to this therapy as well (108). A recent study found that the combination of TMZ and Celecoxib (TMZ 250uM + celecoxib 30uM) inhibited GBM resistance to chemotherapy (48). Interestingly, studies have also found that regular long-time use of NSAIDs reduces risk for GBM in adult populations (114).

However, many COX2 inhibitors have been removed from use and clinical trials have been cut short due to increased knowledge and FDA warnings regarding toxic effects on both the cardiovascular and gastrointestinal system, as well as increased risk for MI and strokes (108, 115). PGE2 terminal synthases and EP receptors, which are downstream in the Cox-2-Id1 axis and induce Id1 and resulting radioresistance (109), have been suggested as possible alternative therapeutic targets (116).

### 4.5. CCL2/CCR2 inhibitors

The CCL2–CCR2 axis has been known to increase levels of Myeloid derived suppressor cells (MDSCs) within GBM microenvironment, which increase GBM immunosuppression (117). MDSCs navigate the tumor microenvironment through chemokine receptors 2 (CCR2); GBM tumors express two CCR2 ligands, CCL2 and CCL7, which cause the tumor to signal the infiltration of CCR2+ cells (118). Therefore, CCL2/CCR2 inhibitors have been thought of as possible therapeutic targets for treating GBM.

A recent study illustrated that CCR2 inhibition using the orally available small molecule inhibitor of CCR2 CCX872 in murine glioma models reduced MDSC tumor infiltration by sequestering these CCR2+ cells within the bone marrow; this ultimately resulted in the reversal of GBM resistance to anti-PD-1 therapies and increased median survival alone and median and OS when used combinatorically with anti-PD-1 therapy (119). Additionally, a recent study illustrated that CCL2 affects the Wnt/β-catenin pathway, and that knockout of CCL2 and β-catenin separately caused a decrease in monocyte infiltration and GSCs proliferation (120).

TAMs, which rely on CCL2, promote angiogenesis through increasing VEGF, and therefore aid in GBM resistance to bevacizumab, an anti-angiogenic therapy (121). The CCL2 inhibitor, mNOX-E36, was shown to decrease TAM recruitment, angiogenesis, and tumor volume in a rat model of GBM (121).

### 4.6. IL6 inhibitors

A recent study showed that IL-6 inhibition or blockade decreased macrophage infiltration modestly by reducing CD40 expression in TAMs, but did not increase the efficiency of PD-1 and CTLA-4 checkpoint inhibitors (122). However, the same study found that the combination of IL-6 inhibitors and CD40 agonists do successfully reduce GBM resistance to immune-checkpoint therapy, but that the triple therapy (CD40 antibody, IL-6 antibody, and ICIs) is required for tumor regression and increased median survival in mice (21 days to 37 days) (122). This proposed triple therapy illustrates the importance of combinatorial therapies and increases the validity for further studies investigating how combinatorial therapies might reverse resistant GBMs.

### 4.7. GITR inhibitors

Treg cells contribute to both GBM and rGBM resistance to ICI therapies by infiltrating the tumor microenvironment and limiting the cytotoxic CD8 T lymphocytes anti-tumor activities (123). Treg cells constitutively express Glucocorticoid-induced tumor necrosis factor related protein (GITR); Anti-GITR agonistic antibody therapy has gained attention as a therapeutic target in GBM due to the success of the therapy in other cancer models, such as bladder cancers in mice (1). In a study involving a GBM model, survival was increased after αGITR therapy in combination with stereotactic radiation (55). To build on those results, a recent study used murine models of GBM and illustrated that αGITR treatment decreased resistance to αPD1 and transitioned the immunosuppressive Treg cells to CD4 T cells with anti-tumor properties (124). Further, these results illustrate the benefit of targeting Treg cells due to their adaptive large population within the tumor microenvironment.

In a separate similar study, investigators found that the administration of anti-GITR monotherapy in mice with GL261 tumors resulted in increased overall survival of the mice as well as the dendritic cell population, but that the success was dependent on the location of therapy injection, with optimal results occurring when injection was at the core of the glioma compared to peripheral injection (125). These promising results were due to a decrease in Treg expression of granzyme B (GrB) as well as Treg selective reduction (125). This illustrated the promising impact of αGITR therapy and the importance of injection sites in the success rate, and underscores the importance of further investigation into the safety and efficacy of these therapies within human GBM.

### 4.8. CAN-2409 therapy

CAN-2409, a non-replicating adenovirus that encodes herpes simplex virus (HSV) thymidine kinase (tk), is an oncolytic viral immunotherapy that induces both a local and a systemic immune response in solid tumors, as well as induces tumor cell death when combined with ganciclovir (GCV) or valacyclovir (86, 126). CAN-2409 induces DNA damage by activating pro-drug ganciclovir (GCV) to GCV triphosphate which has nucleoside analog properties that result in apoptosis (86, 127). It should be highlighted that CAN-2409 therapeutic efficacy is substantially diminished when used in conjunction with continuous high-dose dexamethasone (86).

CAN-2409 is considered a promising therapy for GBM. In a phase I clinical trial, it was shown that CAN-2409 significantly increased OS when used in conjunction with SOC (87). Additionally, triple therapy of SOC + CAN-2409 + anti-PD-1 induced intratumoral T cell infiltration and increased OS in animal models (128). A recent study investigated the combinatorial treatment of DNAdamage-response inhibitor ATR inhibitor AZD6738 and CAN-2409 within a murine glioma model (86, 126). While long-term immunity was not increased, the investigators found that the combinatorial therapy increased overall survival compared to CAN-2409 alone (66.7% to 50%) by increasing DNA damage (86). These results illustrate how combinatorial treatments may improve CAN-2409 therapeutic efficacy.

### 4.9. Targeting GSC through discrimination of subtypes

Of note, a recent study provides a novel machine-learning stemness-based model that discriminates GSC subtypes and their differing responses to immunotherapies, highlighting patient populations that may have an increased response to immunotherapies than others (89). The same study found that patients in Stemness Subtype I, characterized by a higher load burden of somatic mutations and copy number alterations, were both more *responsive* to immunotherapies such as anti-PD1 treatments, as well as more *resistant* to the chemotherapy temozolomide (89). Although this study only looked at genomic alterations, it is expected that epigenetic variability (histone, transcriptome, proteome) will also impact glioblastoma responsiveness to a multitude of immune and chemotherapies. This further underscores the increasing evidence in support of personalized combinatorial therapies that include considerations for the various individual tumor characteristics of each GBM patient.

## 5. Conclusion

Although immunotherapies have revolutionized the treatment of hematological malignancies, there has not been the same level of success in glioblastoma due to multiple avenues of intrinsic and adaptive immune resistance. Due to the inter and intra-tumoral heterogeneity found across and within GBM tumors, there has been great interest in researching targeted therapies that specifically address characteristics of individual GBM tumors to increase survival. Immune mechanism which can target multiple epitopes, such as the dendritic cell based therapeutic vaccine DCVax, will still need to overcome the immunosuppressive factors described in this review. Additionally, combinatorial therapies alongside maximal resection, radiation therapy, and TMZ are essential to successfully target multiple mechanisms of therapy resistance. Future studies should continue to deepen the current understanding of GBM therapy resistant mechanisms to identify new potential therapy targets as well as to identify the appropriate immunotherapy adjuncts in order to continue to improve prognosis.

## Author contributions

EW, ST, and RD: conceptualization. EW and JG: writing—original draft. ST, RD, and EW: writing—review and editing. ST: supervision. All authors contributed to the article and approved the submitted version.

## Funding

This article was supported by the Department of Neurosurgery, Warren Alpert Medical School, Brown University.

## Conflict of interest

The authors declare that the research was conducted in the absence of any commercial or financial relationships that could be construed as a potential conflict of interest.

## Publisher’s note

All claims expressed in this article are solely those of the authors and do not necessarily represent those of their affiliated organizations, or those of the publisher, the editors and the reviewers. Any product that may be evaluated in this article, or claim that may be made by its manufacturer, is not guaranteed or endorsed by the publisher.
