# Supplementary material for: Barriers to overcoming immunotherapy resistance in glioblastoma
Source: Front Med (Lausanne). 2023 May 18;10:1175507. doi: 10.3389/fmed.2023.1175507 (PMC10232794; doi:10.3389/fmed.2023.1175507)
Supplement: Supplementary file 1 [file Data_Sheet_1.pdf]

Supplementary Materials

Barriers to overcoming immunotherapy resistance in glioblastoma

Julia S. Gillette[1], Elaina J. Wang[1], Richard S. Dowd[1], Steven A. Toms[1*]

[1]Departments of Neurosurgery, The Warren Alpert Medical School of Brown University, Providence, Rhode Island

*** Correspondence:**
Steven A. Toms
steven_toms@brown.edu

1 Supplementary Table 1[1]

Mechanisms of Immunotherapy Resistance in Glioblastoma

Mechanisms of Immunotherapy Resistance in Glioblastoma	Example	Reference
**Intrinsic (Primary) Resistance**<br>Immunosuppression	CNS immune privilege / Blood Brain Barrier	22, 30, 32-41
Inter and intra-tumoral heterogeneity	1) Transcriptional diversity: oncogenic signaling, proliferation, immune signaling, and angiogenesis<br>2) phenotype changes : histone methylation, transcriptome, RNA methylation, and protein alteration<br>3) Invasive edge cells that secrete VEGF and IL-8 and over-express HIF-1	42-60
Immune dysfunction	1) T-cell dysfunction<br>2) tumor-associated macrophages (TAMs), microglia (MG) , and monocyte-derived macrophages (MDMs) populations	1,2,15,18, 61-66
**Adaptive (Secondary) Resistance**<br>Selection of resistant intratumoral populations and changes in recurrent glioblastoma	1) "tumor microtubes" to accomplish invasion, proliferation, and repopulation after treatment<br>2) plasticity of glioblastoma stem-like cells (GSCs)<br>3) treatment-resistant state via IL13Rα2 antigen loss GBM variants, up-regulation of IDO1, PD-L1, and IL-10, plasticity and antigen drift	12, 67-82
Effect of steroids on immune response	High-dose steroid therapy (dexamethasone): CCR7 expression loss in T cells, reduced OS in TTF-treated GBM patients, decreases the efficacy of viral oncolytic immunotherapy, CAN-2409	26, 83-89

2 Supplementary Table 2[1]*

Ongoing Immunotherapy Clinical Trials for Glioblastoma

Data Extracted from **Home - ClinicalTrials.gov*. (n.d.). Retrieved April 3, 2023, from https://www.clinicaltrials.gov/

**Ongoing Immunotherapy Clinical Trials**	**Intervention/ Treatment**	**Trial Title**	**Phase**	**Clinical trial Identifier / Reference**
**Ongoing Checkpoint Inhibitor Clinical Trials**	**Drug**: Pembrolizumab	Pembrolizumab (MK-3475) in Patients With Recurrent Malignant Glioma With a Hypermutator Phenotype	Not Applicable	NCT02658279
	**Biological**: Ipilimumab<br>**Procedure**: MRI<br>**Biological**: Nivolumab	A Study Testing the Effect of Immunotherapy (Ipilimumab and Nivolumab) in Patients With Recurrent Glioma With Elevated Mutational Burden	Phase 2	NCT04145115
	**Drug**: Epacadostat<br>**Drug**: Bevacizumab,<br>**Radiation**: Radiation therapy<br>**Procedure**: Peripheral blood draw<br>**Drug**: Retifanlimab	Retifanlimab and Epacadostat in Combination With Radiation and Bevacizumab in Patients With Recurrent Gliomas	Phase 2	NCT03532295
	**Drug**: Pembrolizumab<br>**Drug**: Bevacizumab,<br>**Radiation**: Re-irradiation	Pembrolizumab and Reirradiation in Bevacizumab Naïve and Bevacizumab Resistant Recurrent Glioblastoma	Phase 2	NCT03661723
	**Biological**: MK-3475<br>**Device**: MRI-guided laser ablation<br>**Procedure**: Surgical resection/debulking, biopsy, blood draw for research purposes, cervical lymph node fine needle aspiration	MK-3475 in Combination With MRI-guided Laser Ablation in Recurrent Malignant Gliomas	Phase 1/ Phase 2	NCT02311582
	**Drug**: Pembrolizumab at 7 days prior<br>**Drug**: Pembrolizumab at 14 days post<br>**Drug**: Pembrolizumab at 35 days post<br>**Procedure**: Laser Interstitial Thermotherapy	Laser Interstitial Thermotherapy (LITT) Combined With Checkpoint Inhibitor for Recurrent GBM (RGBM)	Phase 1/ Phase 2	NCT03277638
**Ongoing (CAR) T-Cell Therapy Clinical Trials**	**Drug**: B7-H3 CAR-T<br>**Drug**: Temozolomide<br>**Molecular Target:** B7-H3	Pilot Study of B7-H3 CAR-T in Treating Patients With Recurrent and Refractory Glioblastoma	Phase 1	NCT04385173
	**Drug**: Temozolomide,<br>**Biological**: B7-H3 CAR-T<br>**Molecular Target:** B7-H3	B7-H3 CAR-T for Recurrent or Refractory Glioblastoma	Phase 1/ Phase 2	NCT04077866
	**Biological**: CD147-CART<br>**Molecular Target**: CD147	CD147-CART Cells in Patients With Recurrent Malignant Glioma.	Early Phase 1	NCT04045847

	**Genetic**: (C7R)-GD2.CART cells<br>**Molecular Target**: GD2	C7R-GD2.CAR T Cells for Patients With GD2-expressing Brain Tumors (GAIL-B)	Phase 1	NCT04099797
	**Biological**: CART-EGFRvIII T cells<br>**Biological:** Pembrolizumab<br>**Molecular Target:** EGFRvIII	CART-EGFRvIII + Pembrolizumab in GBM	Phase 1	NCT03726515
	**Biological**: EGFRvIII-CARs<br>**Molecular Target:** EGFRvIII	Intracerebral EGFR-vIII CAR-T Cells for Recurrent GBM (INTERCEPT)	Phase 1	NCT03283631
	**Biological**: IL13Ralpha2-specific Hinge-optimized 4-1BB-co-stimulatory CAR/Truncated CD19-expressing Autologous TN/MEM Cells<br>**Biological**: IL13Ralpha2-specific Hinge-optimized 41BB-co-stimulatory CAR Truncated CD19-expressing Autologous T-Lymphocytes<br>**Other**: Laboratory Biomarker Analysis<br>**Procedure**: MRI<br>**Procedure**: Magnetic Resonance Spectroscopic Imaging<br>**Other**: Quality-of-Life Assessment<br>**Molecular Target**: IL13Ra2	Genetically Modified T-cells in Treating Patients With Recurrent or Refractory Malignant Glioma	Phase 1	NCT02208362
	**Biological**: IL13Ralpha2-specific Hinge-optimized 4-1BB-co-stimulatory CAR/Truncated CD19-expressing Autologous TN/MEM Cells<br>**Biological**: Ipilimumab,<br>**Biological**: Nivolumab<br>**Molecular Target**: IL13Ra2	IL13Ra2-CAR T Cells With or Without Nivolumab and Ipilimumab in Treating Patients With GBM	Phase 1	NCT04003649
	**Biological**: Chlorotoxin (EQ)-CD28-CD3zeta-CD19t-expressing CAR T-lymphocytes (ICT delivery)<br>**Biological**: Chlorotoxin (EQ)-CD28-CD3zeta-CD19t-expressing CAR T-lymphocytes (via ICT/ICV dual delivery)<br>**Molecular Target:** MMP2 (Chlorotoxin)	Chimeric Antigen Receptor (CAR) T Cells With a Chlorotoxin Tumor-Targeting Domain for the Treatment of MMP2+ Recurrent or Progressive Glioblastoma	Phase 1	NCT04214392
	**Biological**: chimeric antigen receptor T cells	Personalized Chimeric Antigen Receptor T Cell Immunotherapy for Patients With Recurrent Malignant Gliomas	Phase 1	NCT03423992

**Ongoing Vaccination Clinical Trials**	ADU-623	Phase I Study of Safety and Immunogenicity of ADU-623, a Live-attenuated Listeria Monocytogenes Strain (ΔactA/ΔinlB) Expressing the EGFRvIII-NY-ESO-1 Vaccine, in Patients With Treated and Recurrent WHO Grade III/IV Astrocytomas	Phase 1	NCT01967758
	**Biological**: HSPPC-96<br>**Drug**: Temozolomide<br>**Procedure**: Standard Surgical Resection	PHASE 2, Multi-center, Single Arm Investigation of HSPPC-96 Vaccine With Temozolomide in Patients With Newly Diagnosed Glioblastoma Multiforme	Phase 2	NCT00905060
	HSPPC-96,<br>**Biological**: gp96	Research for Immunotherapy of Glioblastoma With Autologous Heat Shock Protein gp96	Phase 1	NCT02122822
	**Biological**: HSPPC-96<br>**Drug**: bevacizumab	A Phase II Randomized Trial Comparing the Efficacy of Heat Shock Protein-Peptide Complex-96 (HSPPC-96) (NSC #725085, ALLIANCE IND # 15380) Vaccine Given With Bevacizumab Versus Bevacizumab Alone in the Treatment of Surgically Resectable Recurrent Glioblastoma Multiforme (GBM)	Phase 2	NCT01814813
	**Drug**: Pembrolizumab<br>**Biological**: HSPPC-96<br>**Drug**: Temozolomide<br>**Other**: Placebo	A Randomized, Double Blind Phase II Trial of Surgery, Radiation Therapy Plus Temozolomide and Pembrolizumab With and Without HSPPC-96 in Newly Diagnosed Glioblastoma (GBM)	Phase 2	NCT03018288
	**Drug**: IDH1 peptide vaccine	Targeting IDH1R132H in WHO Grade III-IV IDH1R132H-mutated Gliomas by a Peptide Vaccine - a Phase I Safety, Tolerability and Immunogenicity Multicenter Trial (NOA-16)	Phase 1	NCT02454634
	**Biological**: K27M peptide<br>**Drug**: Nivolumab	H3.3K27M Specific Peptide Vaccine Combined With Poly-ICLC With and Without PD-1 Inhibition Using Nivolumab for the Treatment of Newly Diagnosed HLA-A2 (02:01)+ H3.3K27M Positive Diffuse Intrinsic Pontine Glioma (DIPG) and Newly Diagnosed HLA-A2 (02:01)+ H3.3K27M Positive Gliomas	Phase 1/ Phase 2	NCT02960230
	**Other**: Laboratory Biomarker Analysis<br>**Drug**: Montanide ISA 51 VG<br>**Biological**: Sargramostim<br>**Biological**: SVN53-67/M57-KLH Peptide Vaccine<br>**Drug**: Temozolomide	A Phase II Study of the Safety and Efficacy of SVN53-67/M57-KLH (SurVaxM) in Survivin-Positive Newly Diagnosed Glioblastoma	Phase 2	NCT02455557
	DCs vaccine (DCVax)	A Phase III Clinical Trial Evaluating DCVax®-L, Autologous Dendritic Cells Pulsed With Tumor Lysate Antigen For The Treatment Of Glioblastoma Multiforme (GBM)	Phase 3	NCT00045968

	**Biological**: BTSC mRNA-loaded DCs	Recurrent GBM Stem Cell Tumor Amplified RNA Immunotherapy Trial	Phase 1	NCT00890032
	**Biological**: Dendritic Cells<br>**Drug**: Imiquimod	Phase I Study of Vaccination With Dendritic Cells Loaded With Brain Tumor Stem Cells for Recurrent or Progressive Malignant Gliomas	Phase 1	NCT01171469
	**Biological**: DEC-205/NY-ESO-1 Fusion Protein CDX-1401<br>**Other**: Laboratory Biomarker Analysis<br>**Other**: Pharmacological Study<br>**Drug**: Sirolimus	A Phase I Clinical Trial of mTOR Inhibition With Rapamycin for Enhancing Intranodal Dendritic Cell Vaccine Induced Anti-tumor Immunity in Patients With NY-ESO-1 Expressing Solid Tumors	Phase 1	NCT01522820
	**Biological**: tetanus toxoid<br>**Biological**: therapeutic autologous dendritic cells<br>**Biological**: therapeutic autologous lymphocytes	Anti-Tumor Immunotherapy Targeted Against Cytomegalovirus in Patients With Newly-Diagnosed Glioblastoma Multiforme During Recovery From Therapeutic Temozolomide-induced Lymphopenia	Phase 1	NCT00639639
	**Biological**: pp65-shLAMP DC with GM-CSF<br>**Biological**: unpulsed PBMC and saline<br>**Drug**: Td<br>**Drug**: Saline<br>**Biological**: pp65-flLAMP DC with GM-CSF	A Phase II Randomized, Blinded, and Placebo-controlled Trial of CMV RNA-Pulsed Dendritic Cells With Tetanus-Diphtheria Toxoid Vaccine in Patients With Newly-Diagnosed Glioblastoma	Phase 2	NCT02465268
	**Biological**: Unpulsed DCs<br>**Biological**: Td<br>**Biological**: Human CMV pp65-LAMP mRNA-pulsed autologous DCs<br>**Biological**: 111In-labeled DCs<br>**Drug**: Temozolomide<br>**Drug**: Saline<br>**Drug**: Basiliximab	Evaluation of Overcoming Limited Migration and Enhancing Cytomegalovirus-specific Dendritic Cell Vaccines With Adjuvant TEtanus Pre-conditioning in Patients With Newly-diagnosed Glioblastoma	Phase 2	NCT02366728
	**Biological**: autologous tumor lysate-pulsed DC vaccination<br>**Biological**: Tumor lysate-pulsed DC vaccination+0.2% resiquimod<br>**Biological**: Tumor-lysate pulsed DC vaccination +adjuvant polyICLC	A Phase II Clinical Trial Evaluating Autologous Dendritic Cells Pulsed With Tumor Lysate Antigen +/- Toll-like Receptor Agonists for the Treatment of Malignant Glioma	Phase 2	NCT01204684
	**Biological**: RNA-loaded dendritic cell vaccine<br>**Drug**: basiliximab	REGULATory T-Cell Inhibition With Basiliximab (Simulect®) During Recovery From Therapeutic Temozolomide-induced Lymphopenia During Antitumor Immunotherapy Targeted Against Cytomegalovirus in Patients With Newly-Diagnosed Glioblastoma Multiforme	Phase 1	NCT00626483

	**Other**: Laboratory Biomarker Analysis<br>**Biological**: Malignant Glioma Tumor Lysate-Pulsed Autologous Dendritic Cell Vaccine<br>**Drug**: Temozolomide	Pilot Clinical Trial of Allogeneic Tumor Lysate-Pulsed Autologous Dendritic Cell Vaccination in Newly Diagnosed Glioblastoma	Phase 1	NCT01957956
	**Biological**: ICT-121 DC vaccine	Immunological Targeting of CD-133 in Recurrent Glioblastoma: A Multi-center Phase I Translational and Clinical Study of an Autologous CD-133 DC Vaccine	Phase 1	NCT02049489
	**Biological**: Dendritic Cell Vaccine<br>**Biological**: Tumor Lysate<br>**Drug**: Imiquimod,<br>**Procedure**: Leukapheresis	Dendritic Cell Vaccine For Malignant Glioma and Glioblastoma Multiforme in Adult and Pediatric Subjects	Phase 1	NCT01808820
	**Biological**: Dendritic cell vaccination + temozolomide chemotherapy + involved field radiation therapy<br>**Biological**: Dendritic cell vaccination, + optional bevacizumab treatment	Phase I Trial of Vaccination With Autologous Dendritic Cells Pulsed With Lysate Derived From an Allogeneic Glioblastoma Stem-like Cell Line for Patients With Newly Diagnosed or Recurrent Glioblastoma	Phase 1	NCT02010606
	**Biological**: SL-701; poly-ICLC (polyinosinic-polycytidylic acid stabilized with polylysine and carboxymethyl cellulose)<br>**Drug**: Bevacizumab	A Phase 1/2 Study of SL-701, a Subcutaneously Injected Multivalent Glioma-Associated Antigen Vaccine, in Adult Patients With Recurrent Glioblastoma Multiforme (GBM)	Phase 1/ Phase 2	NCT02078648
	**Biological**: IMA 950Biological: Poly ICLC<br>**Other**: Immunomonitoring	Phase I/II Study of Intradermal IMA950 Peptide-based Vaccine Adjuvanted With Intra Muscular Poly-ICLC in Combination With Temozolomide in Newly Diagnosed HLA-A2 Glioblastoma Patients	Phase 1/ Phase 2	NCT01920191
	**Biological**: glioblastoma multiforme multipeptide vaccine IMA950<br>**Biological**: sargramostim,<br>**Drug**: temozolomide<br>**Other**: laboratory biomarker analysis<br>**Other**: pharmacological study<br>**Procedure**: adjuvant therapy<br>**Radiation**: radiation therapy	A Cancer Research UK Phase I Trial of IMA950 (A Novel Multi-Peptide Vaccine) Plus GM-CSF in Patients With Newly Diagnosed Glioblastoma	Phase I	NCT01222221
	**Drug**: Bevacizumab<br>**Drug**: Rindopepimut (CDX-110) with GM-CSFDrug: KLH	A Phase II Study of Rindopepimut/GM-CSF in Patients With Relapsed EGFRvIII-Positive Glioblastoma	Phase 2	NCT01498328
	**Drug**: CDX-110 with GM-CSF<br>**Drug**: Temozolomide	A Phase II Study of CDX-110 With Radiation and Temozolomide in Patients With Newly Diagnosed Glioblastoma Multiforme	Phase 2	NCT00458601

	**Drug**: Rindopepimut (CDX-110) with GM-CSF<br>**Drug**: Temozolomide<br>**Drug**: KLH	An International, Randomized, Double-Blind, Controlled Study of Rindopepimut/GM-CSF With Adjuvant Temozolomide in Patients With Newly Diagnosed, Surgically Resected, EGFRvIII-positive Glioblastoma	Phase 3	NCT01480479
	**Biological**: PEP-3 vaccine<br>**Biological**: sargramostim<br>**Drug**: Temozolomide	A Complementary Trial of an Immunotherapy Vaccine Against Tumor-Specific EGFRvIII	Phase 2	NCT00643097
	**Biological**: Dendritic vaccine pulsed with multiple peptides<br>**Biological**: First booster vaccine phase<br>**Biological**: Second booster vaccine phase	A Phase I/II Evaluation of Vaccination With Type 1 Dendritic Cells Pulsed With Multiple Peptides in the Treatment of HLA-A2 Positive Patients With Recurrent Malignant Gliomas	Phase 1/ Phase 2	NCT00766753
	**Biological**: SL-701; poly-ICLC (polyinosinic-polycytidylic acid stabilized with polylysine and carboxymethyl cellulose)<br>**Drug**: Bevacizumab	A Phase 1/2 Study of SL-701, a Subcutaneously Injected Multivalent Glioma-Associated Antigen Vaccine, in Adult Patients With Recurrent Glioblastoma Multiforme (GBM)	Phase 1/ Phase 2	NCT00293423
**Ongoing Oncolytic Virotherapy Clinical Trials**	**Drug**: Biological G207	Phase II Clinical Trial of HSV G207 With a Single 5 Gy Radiation Dose in Children With Recurrent High-Grade Glioma	Phase 2	NCT04482933
	**Biological**: DNX-2401,<br>**Biological**: pembrolizumab	Combination Adenovirus + Pembrolizumab to Trigger Immune Virus Effects (CAPTIVE)	Phase 2	NCT02798406
	**Biological**: PVSRIPO	A Multicenter Phase 2 Study of Oncolytic Polio/Rhinovirus Recombinant (PVSRIPO) in Recurrent WHO Grade IV Malignant Glioma Patients	Phase 2	NCT02986178
	**Biological**: lerapolturev,<br>**Biological**: pembrolizumab	A Phase 2, Open-label, Single-arm Study Evaluating the Efficacy, Safety and Tolerability of Lerapolturev (PVSRIPO) and the Immune Checkpoint Inhibitor Pembrolizumab in the Treatment of Patients With Recurrent Glioblastoma	Phase 2	NCT04479241
	**Drug**: DNX-2401,<br>**Procedure**: Tumor Removal	Phase I Trial of Conditionally Replication-Competent Adenovirus (DNX-2401, Formerly Known as Delta-24-RGD-4C) for Recurrent Malignant Gliomas	Phase 1	NCT00805376
	**Biological**: delta-24-RGD adenovirus	A Phase I/II Trial of a Conditionally Replication-competent Adenovirus (Delta-24-rgd) Administered by Convection Enhanced Delivery in Patients With Recurrent Glioblastoma	Phase 1/ Phase 2	NCT01582516
	**Procedure**: DNX2401 and Temozolomide	Phase I Trial of Combination of DNX-2401 (Formerly Named Delta-24-RGD) Oncolytic Adenovirus With a Short Course of Temozolomide for Treatment of Glioblastoma at First Recurrent	Phase 1	NCT01956734

	**Drug**: Single intratumoral injection of DNX-2401<br>**Drug**: Interferon-gamma	A Phase 1b, Randomized, Multi-center, Open-label Study of a Conditionally Replicative Adenovirus (DNX-2401) and Interferon Gamma (IFN-γ) for Recurrent Glioblastoma or Gliosarcoma (TARGET-I)	Phase 1	NCT02197169
	**Drug**: DNX-2440 injection	Phase I Trial of DNX-2440 Oncolytic Adenovirus in Patients With Recurrent Glioblastoma	Phase 1	NCT03714334
	**Biological**: Oncolytic Adenovirus Ad5-DNX-2401,<br>**Procedure**: Therapeutic Conventional Surgery	Phase I Clinical Trial of Allogeneic Bone Marrow Human Mesenchymal Stem Cells Loaded With A Tumor Selective Oncolytic Adenovirus, DNX-2401, Administered Via Intra-Arterial Injection in Patients With Recurrent High-Grade Glioma	Phase 1	NCT03896568
	**Drug**: G207, an oncolytic virus	An Open-Label Phase Ib/II Study of the Safety, Tolerability and Efficacy of G207, a Genetically Engineered Herpes Simplex Type-1 Virus, Administered Intracerebrally to Patients With Recurrent Malignant Glioma	Phase 1/ Phase 2	NCT00036699
	**Drug**: G207	A Staged Phase 1 Study of the Treatment of Malignant Glioma With G207, a Genetically Engineered HSV-1, Followed by Radiation Therapy	Phase 1	NCT00157703
	**Biological**: M032 (NSC 733972)	A Phase 1 Study of M032 (NSC 733972), a Genetically Engineered HSV-1 Expressing IL-12, in Patients With Recurrent/Progressive Glioblastoma Multiforme, Anaplastic Astrocytoma, or Gliosarcoma	Phase 1	NCT02062827
	**Biological**: G207	Phase I Clinical Trial of HSV G207 Alone or With a Single Radiation Dose in Children With Recurrent Supratentorial Brain Tumors	Phase 1	NCT02457845
	**Drug**: rQNestin, Drug: Cyclophosphamide,<br>**Procedure**: Stereotactic biopsy	A Phase I Study of the Treatment of Recurrent Malignant Glioma With rQNestin34.5v.2, a Genetically Engineered HSV-1 Virus, and Immunomodulation With Cyclophosphamide	Phase 1	NCT03152318
	**Biological**: G207	Phase 1 Trial of Engineered HSV G207 in Children With Recurrent or Refractory Cerebellar Brain Tumors	Phase 1	NCT03911388
	**Biological**: C134	A Phase I Trial of IRS-1 HSV C134 Administered Intratumorally in Patients With Recurrent Malignant Glioma	Phase 1	NCT03657576
	**Biological**: Carcinoembryonic Antigen-Expressing Measles Virus,<br>**Other**: Laboratory Biomarker Analysis,<br>**Procedure**: Therapeutic Conventional Surgery	Phase I Trial of a Measles Virus Derivative Producing CEA (MV-CEA) in Patients With Recurrent Glioblastoma Multiforme (GBM)	Phase 1	NCT00390299

	**Biological**: Recombinant nonpathogenic polio-rhinovirus chimera (PVSRIPO)	Dose-finding and Safety Study of an Oncolytic Polio/Rhinovirus Recombinant Against Recurrent WHO Grade IV Malignant Glioma	Phase 1	NCT01491893
	**Biological**: Polio/Rhinovirus Recombinant (PVSRIPO)	Phase Ib Study of Oncolytic Polio/Rhinovirus Recombinant Against Recurrent Malignant Glioma in Children	Phase 1	NCT03043391
	**Drug**: H-1PV	Phase I/IIa Study of Intratumoral/Intracerebral or Intravenous/Intracerebral Administration of Parvovirus H-1 (ParvOryx) in Patients With Progressive Primary or Recurrent Glioblastoma Multiforme.	Phase 1/ Phase 2	NCT01301430
	**Biological**: REOLYSIN®	A Phase I/II Clinical Trial to Evaluate Dose Limiting Toxicity and Efficacy of Intralesional Administration of REOLYSIN® for the Treatment of Patients With Histologically Confirmed Recurrent Malignant Gliomas	Phase 1	NCT00528684
	**Other**: Laboratory Biomarker Analysis **Biological**: Sargramostim, **Biological**: Wild-type Reovirus	Phase 1 Study of Replication Competent Reovirus (Reolysin®) in Combination With GM-CSF in Pediatric Patients With Relapsed or Refractory Brain Tumors	Phase 1	NCT02444546
	**Drug**: Combination of TG6002 and 5-flucytosine (5-FC, Ancotil®)	Safety and Efficacy of the ONCOlytic VIRus Armed for Local Chemotherapy, TG6002/5-FC, in Recurrent Glioblastoma Patients	Phase 1/ Phase 2	NCT03294486

References

1. U.S. National Library of Medicine. **ClinicalTrials.gov background**. U.S. National Library of Medicine, Bethesda, MD (2014). Available at https://clinicaltrials.gov/ct2/about-site/background . Accessed April 20th, 2023
